# Surgery increases cell death and induces changes in gene expression compared with anesthesia alone in the developing piglet brain

**DOI:** 10.1371/journal.pone.0173413

**Published:** 2017-03-29

**Authors:** Kevin D. Broad, Go Kawano, Igor Fierens, Eridan Rocha-Ferreira, Mariya Hristova, Mojgan Ezzati, Jamshid Rostami, Daniel Alonso-Alconada, Badr Chaban, Jane Hassell, Bobbi Fleiss, Pierre Gressens, Robert D. Sanders, Nicola J. Robertson

**Affiliations:** 1 Institute for Women’s Health, University College London, London, United Kingdom; 2 Centre for the Developing Brain, Kings College, St Thomas Campus, London, United Kingdom; 3 Inserm, U1141, Paris, France; 4 University Paris Diderot, Sorbonne Paris Cite, UMRS 1141, Paris, France; 5 Department of Anesthesiology, University of Wisconsin, Madison, Wisconsin, United States of America; Universidade de Sao Paulo, BRAZIL

## Abstract

In a range of animal species, exposure of the brain to general anaesthesia without surgery during early infancy may adversely affect its neural and cognitive development. The mechanisms mediating this are complex but include an increase in brain cell death. In humans, attempts to link adverse cognitive development to infantile anaesthesia exposure have yielded ambiguous results. One caveat that may influence the interpretation of human studies is that infants are not exposed to general anaesthesia without surgery, raising the possibility that surgery itself, may contribute to adverse cognitive development. Using piglets, we investigated whether a minor surgical procedure increases cell death and disrupts neuro-developmental and cognitively salient gene transcription in the neonatal brain. We randomly assigned neonatal male piglets to a group who received 6h of 2% isoflurane anaesthesia or a group who received an identical anaesthesia plus 15 mins of surgery designed to replicate an inguinal hernia repair. Compared to anesthesia alone, surgery-induced significant increases in cell death in eight areas of the brain. Using RNAseq data derived from all 12 piglets per group we also identified significant changes in the expression of 181 gene transcripts induced by surgery in the cingulate cortex, pathway analysis of these changes suggests that surgery influences the thrombin, aldosterone, axonal guidance, B cell, ERK-5, eNOS and GABA_A_ signalling pathways. This suggests a number of novel mechanisms by which surgery may influence neural and cognitive development independently or synergistically with the effects of anaesthesia.

## Introduction

There is considerable debate concerning the possibility that exposure of the developing brain to general anesthesia may adversely affect cognitive development. Well controlled studies in a range of animal species (including piglets) have provided clear evidence that early developmental exposure to general anesthesia, without surgery leads to increases in cell death, reductions in neuro and synapto-genesis, a disruption to the expression of cognitively salient genes and deficits in cognitive function that persist throughout life [1–10]. In humans, attempts to definitively link cognitive dysfunction to infantile anesthetic exposure have yielded ambiguous results. Several studies have suggested that exposure to general anesthesia and surgery is associated with an increased risk of adverse cognitive development, a risk which increases following protracted or multiple procedures [11–14]. However, other studies have concluded that there is no association between anesthetic exposure and adverse cognitive development [14–16]. One potential confounder, of human clinical studies, that affects their interpretation is that infants undergo both general anesthesia and surgery, which makes the dissociation of their potential effects impossible. Another is that infants who undergo anesthesia and surgery are usually compared with healthy controls, raising the possibility that the data derived from the anesthesia and surgery cohorts, is reflective of the fact that infants who require surgery early in life have underlying medical or developmental problems that may be associated with a vulnerability to cognitive dysfunction [15]. The recent GAS consortium study [17] avoided these two potential confounders by using randomised infants (approx 60 weeks of age) with an identical inguinal hernia repair supported by either conscious regional or servoflurane based general anesthesia. Although extrapolation of cognitive function at 2 years to that in adulthood is problematic, this important study suggested that exposure to a single episode of servoflurane anesthesia (median of 52 mins) is not associated with an increased risk of adverse cognitive development at this age.

We have recently demonstrated that in neonatal male piglets 6h of exposure to 2% isoflurane exposure (without surgery) induces increases in cell death, microglial activation and disrupts the developmentally appropriate expression of genes supporting neurodevelopment and cognitive function [10]. Our study in conjunction with others in both animals and humans was derived from the hypothesis that the primary deleterious effects of anesthesia and surgery on the developing brain, derive from exposure to anesthesia [1–17]. In contrast to these studies we hypothesised that the experience of surgery itself may contribute to increases in cell death and a disruption to developmentally appropriate gene expression, therefore our study investigated whether a brief surgical procedure compared with anesthesia exposure alone induced: 1) an increase in cell death and 2) significant changes in gene transcription in the developing neonatal piglet brain. In an earlier rodent study, a minor peripheral noxious stimuli exacerbated the cell death induced by anesthesia [18]. We randomly assigned neonatal malepiglets to a control group who received 6h of 2% isoflurane anesthesia (with midolazam pre-sedation, and fentanyl analgesia) or a group who underwent an identical anesthetic treatment plus 15 min of surgery within 1h of the induction of anesthesia, designed to replicate an inguinal hernia repair. The piglet model of anesthesia and surgery used in this study adds a strong pre-clinical aspect to this study, as it allows the surgery to be applied as it would in a clinical setting with extensive physiological support. This support includes regulated ventilation, maintenance of cardiac output, blood volume and electrolytes. At a 6h time point we then assessed brain cell death using terminal deoxynucleotidyl transferase-mediated deoxyuridine-triphosphate (d-UTP) nick-end labelling (TUNEL) and analysed changes in gene transcription in the cingulate cortex using RNAseq.

## Materials and methods

### *In-vivo* procedures

Experiments were approved by the UK Home Office and the UCL Ethics board and performed under UK Home Office Guidelines [Animal (Scientific Procedures) Act, 1986] and EEC directive 86/609/EEC. Twenty four Large White male piglets were obtained from a specialist, isolated, disease free pig breeding facility (location details on request) and randomly assigned to either a control group (n = 12) who received 6h of 2% isoflurane anesthesia (with 0.2mg/ kg-1 midolazam pre-sedation, 3mcg/k/h^-1^ fentanyl analgesia) or a group (n = 12) who received an identical anesthetic treatment plus 15 mins of surgery and closure designed to replicate an inguinal hernia repair within 1h of the induction of anesthesia. Piglets were sedated with midazolam (i.m 0.2mg kg^-1^) and initially received isoflurane 2.5–3% v/v via a facemask prior to endotracheal intubation (Portex endotracheal tube, Smiths Medical, Ashford, Kent, UK), anesthesia was maintained with 2% isoflurane for 6h following line insertion and intubation. The study was designed so the anesthesia regime was equivalent in both control (anesthesia) and treatment group (anesthesia plus surgery) and the anesthesia concentration was maintained at 2% in both groups. No significant blood loss was observed during surgery or closure. Local cutaneous anesthesia was not applied to the surgery site and no movement of the pig was observed when the initial incision was made. Piglets were mechanically ventilated, partial pressure of oxygen (PaO_2_) and carbon dioxide (PaCO_2_) were maintained at 8-13kPa and 4.5–6.5kPa respectively. Fractional inspired oxygen concentrations were maintained at 21% and arterial oxygen saturation was monitored throughout by pulse oximetry (Nonin Medical, Plymouth, MN, UK).

Umbilical arterial and venous catheters were inserted for infusion of maintenance fluids (10% dextrose, 60ml/kg/day), fentanyl (Mercury Pharma, Co Dublin, Eire, 3mcg kg^-1^ h^-1^), antibiotic prophylaxis (single doses of benzylpenicillin 50mg kg^-1^ (Genus Pharma, Berkshire, UK) and gentamicin 2.5mg kg^-1^ (Pathion, Wiltshire, UK)) and to facilitate the continuous monitoring of heart rate and arterial blood pressure. Hourly arterial blood was taken to measure PaO_2_, PaCO_2_, pH, electrolytes, urea, creatinine, glucose, lactate and blood haematocrit (Table 1). Arterial lines were maintained by infusing 0.9% saline solution (0.3ml h^-1^, with sodium heparin (1 IU ml^-1^)). Subjects received continuous physiological monitoring and intensive life support throughout. Infusions of 0.9% saline (10ml kg^-1^) and dopamine (5-15mcg kg^-1^ min^-1^) were given, where necessary, to maintain mean arterial blood pressure >40mmHg.

**Table 1:** Physiological variables compared in neonatal piglets subjected to anesthesia alone (control) and anesthesia and surgery. Body weight, post-natal age and duration of anesthesia were compared at baseline using an unpaired t-test. Physiological parameters were compared at baseline (BL) and at 1h intervals until termination, no statistically significant intergroup differences were observed using a repeated measures ANOVA.

Physiological parameter	Time	Group mean (±SD)	
		Anesthesia	Anesthesia + Surgery
Post-natal age (h)	-	22.58 (8.61)	24.92 (5.12)
Body weight (kg)	-	1.85 (0.16)	1.83 (0.13)
Duration of anaesthesia (h)	-	6.63 (0.17)	6.77 (0.27)
Heart rate (BPM)	BL	140 (17)	139 (13)
	1h	150 (14)	145 (18)
	2h	150 (15)	157 (13)
	3h	148 (20)	158 (14)
	4h	158 (23	161 (15)
	5h	168 (32)	162 (17)
	6h	170 (23)	160 (15)
MABP mmHg	BL	47.8 (5.6)	42.0 (4.6)
	1h	46.2 (6.5)	41.7 (7.5)
	2h	45.9 (6.6)	47.6 (8.5)
	3h	41.5 (7.8)	41.2 (5.2)
	4h	43.7 (11.7)	40.7 (3.7)
	5h	40.5 (5.6)	39.5 (2.5)
	6h	39.6 (5.2)	38.8 (3.3)
Rectal temp °C	BL	38.1 (1.2)	37.9 (1.1)
	1h	38.6 (1.1)	37.6 (1.2)
	2h	38.4 (1.0)	38.3 (1.1)
	3h	38.0 (1.0)	38.5 (0.4)
	4h	38.2 (1.2)	38.5 (0.6)
	5h	38.2 (1.0)	38.4 (0.4)
	6h	38.5 (0.9)	38.4 (0.4)
PaO_2_ kPa	BL	15.8 (8.8)	18.2 (8.5)
	1h	13.2 (4.9)	16.6 (6.8)
	2h	15.2 (7.6)	12.8 (6.6)
	3h	11.3 (3.7)	11.9 (6.5)
	4h	12.8 (3.3)	12.3 (6.7)
	5h	13.3 (4.0)	12.9 (7.4)
	6h	11.5 (2.4)	15.0 (8.2)
PaCO_2_ kPa	BL	4.82 (0.91)	5.14 (1.64)
	1h	6.47 (1.25)	5.28 (1.99)
	2h	6.16 (1.60)	5.32 (0.86)
	3h	6.25 (0.78)	5.93 (1.18)
	4h	6.57 (1.30)	5.90 (0.68)
	5h	5.97 (1.29)	5.52 (1.02)
	6h	6.20 (1.60)	5.38 (1.25)
pH	BL	7.5 (0.1)	7.4 (0.2)
	1h	7.4 (0.9)	7.4 (0)
	2h	7.4 (0.1)	7.4 (0)
	3h	7.4 (0)	7.5 (0.1)
	4h	7.4 (0.1)	7.4 (0.1)
	5h	7.4 (0.1)	7.5 (0.1)
	6h	7.4 (0.1)	7.5 (0.1)
BE mmol/L	BL	8 (4)	6 (6)
	1h	6 (4)	5 (4)
	2h	6 (3)	3 (3)
	3h	6 (3)	5 (4)
	4h	6 (3)	6 (4)
	5h	6 (4)	6 (5)
	6h	6 (4)	7 (5)
Lactate mmol/L	BL	3.6 (1.2)	3.5 (1.7)
	1h	3.3 (1.3)	4.5 (1.9)
	2h	3.8 (2.0)	5.4 (2.5)
	3h	3.9 (1.4)	4.6 (2.2)
	4h	3.6 (1.5)	4.0 (1.9)
	5h	3.4 (1.7)	3.9 (1.8)
	6h	3.3 (1.5)	3.5 (2.0)
Glucose mmol/L	BL	5.0 (1.3)	5.4 (1.0)
	1h	6.6 (1.1)	6.3 (1.0)
	2h	7.3 (0.9)	6.9 (1.0)
	3h	7.5 (1.0)	7.0 (0.9)
	4h	7.2 (1.1)	7.0 (0.8)
	5h	7.4 (1.2)	7.2 (1.2)
	6h	9.6 (0.81)	6.8 (0.7)
Calcium mmol/L	BL	1.4 (0.1)	1.2 (0.5)
	1h	1.5 (0.1)	1.4 (0.1)
	2h	1.5 (0.1)	1.5 (0.1)
	3h	1.4 (0.2)	1.5 (0.1)
	4h	1.5 (0.1)	1.5 (0.1)
	5h	1.4 (0.2)	1.5 (0.1)
	6h	1.5 (0)	1.5 (0.1)

### Assessment of cell death

Piglets were euthanized with pentobarbital, brains perfused with phosphate buffered saline, followed by 4% phosphate buffered paraformaldehyde and post-fixed in 2% paraformaldehyde in PBS (all at pH 7.4 and 4°C) for 10d. The right hemisphere was embedded in paraffin and sectioned (5μm). Representative sections were stained with haematoxylin and eosin (H&E) to assist with the identification of neuroanatomical locations. Cell death was assessed using TUNEL histochemistry, briefly sections were pre-treated for 15mins in 3% hydrogen peroxide, pre-digested with protease K (15mins, 20μg ml^-1^ 65°C, Promega, Southampton, UK), incubated with TUNEL solution (2h, 37°C, Roche, Burgess Hill, UK), and visualized using avidin-biotinylated horseradish complex (ABC, Vector Laboratories, Peterborough, UK) and diaminobenzidine/H_2_O_2_ (DAB, Sigma, Poole, UK). Sections were then dehydrated and cover-slipped with DPX (VWR, Leighton Buzzard, UK). Quantification of cell death was undertaken blind to treatment, for each piglet 2 sections were assessed at Bregma levels 00 and -2.0 (5mm apart), consistency of counting with regard to individual areas was maintained by reference to haematoxylin and eosin stained sections. TUNEL-positive nuclei were counted in three non-overlapping fields of view at x40 magnification (sampling area was 0.075mm^2^ per field of view of view), values pooled converted to cells per mm^2^ and means used for analysis. Cell death was assessed as an overall effect and separately in the cingulate, motor, somatosensory, insula and pyriform cortices, periventricular white matter, internal capsule, caudate nucleus, putamen and thalamus.

### RNA-seq

At euthanasia a fresh, un-perfused biopsy of the right anterior cingulate cortex (adjacent to the sulcus at 5mm posterior to bregma) was taken, placed in RNAlater solution (Qiagen, West Sussex, UK), frozen in liquid nitrogen and stored at -80°C until processing (n = 12 anesthesia, n = 12 anesthesia and surgery). RNA was extracted using the standard protocol for animal tissues supplied with the RNAeasy Midi kit (Qiagen, West Sussex, UK). RNA was assessed using a Nanodrop spectrophotometer (NanoDrop, Wilmington, DE, USA) and Agilent 2100 Bioanalyser (Agilent, Santa Clara, CA, USA) and all samples had a spectral 260/280 ratio of between 2.05–2.13, and a RIN of 9.9–10.

Briefly, mRNA was isolated from total RNA using Oligo dT beads to pull down Poly-Adenylated transcripts. The purified mRNA was fragmented using chemical fragmentation (heat and divalent metal cation) and primed with random hexamers. cDNA was generated using Reverse Transcriptase and random primers. Samples were processed using Illumina’s TruSeq RNA sample prep kit version 2 (p/n RS-122-2001) according to the manufacturer’s instructions, with the following variations in protocol, 250ng total RNA was used as starting material, fragmentation was carried out for 10 instead of 8mins and 12 cycles of PCR were used. The overhanging ends of the cDNA fragments were repaired using an End Repair enzyme mix, this has a 3’ to 5’ exonuclease activity which removes 3’ overhangs and polymerase activity which fills in the 5’ overhangs. The blunt ended cDNA was “A-tailed” at the 3’ end to prevent self-ligation during the addition of the Adaptors (as Adaptors have a complementary “T-tail”). Indexing Adaptors were ligated to the A-Tailed cDNA. These adaptors contain sequences that allow the libraries to be amplified by PCR, bind to the flow cell and be uniquely identified by way of a 6bp Index sequence. Finally, a PCR was carried out to amplify only those cDNA fragments that had adaptors bound to both ends. Libraries to be multiplexed in the same run were pooled in equimolar quantities and calculated from both qPCR and Bioanalyser fragment analysis. Samples were sequenced in a 24-plex pool on a NextSeq 500 instrument (Illumina, San Diego, US) using a 43bp paired end run and an average of 20 million read pairs were generated for each sample. Data was channelled into Genespring GX12 (Agilent, California, USA) and first normalized and summarized using the Robust Multi-array Analysis (RMA) algorithm. Data was filtered, as per normal array analysis protocols, to include only those probe sets falling between the 20^th^ and 100^th^ percentile after normalization. As we used a biologically relevant sample size of 12 piglets per group we employed a threshold of 1.3 fold change and a *p-value* of at least 0.05.

### Statistics

Thresholds for statistical significance was p<0.05. Physical parameters, were compared using repeated measures ANOVA. Saline and dopamine treatment were compared using a Kruskal-Wallis equality of populations rank test. Cell death was compared using an analysis of variance model, overall differences between means and treatment differences for the two treatment groups are presented with 95% C.I.s. Analysis of gene expression was performed using a one-way ANOVA followed by a moderated T-test post-hoc test and a Benjami-Hochberg FDR multiple testing correction and *p*-values calculated asymptotically using endogenous Genespring GX12 software.

## Results

### Physiological parameters are not significantly altered by 15 mins of inguinal surgery

There were no significant intergroup differences in body weight or age. Physiological parameters were controlled to within normal values throughout, with no statistically significant differences in physiological parameters or blood pressure support (saline or dopamine) observed between the two groups (Table 1).

### 15 minutes of surgery increases cell death in the brain

**Fig 1:**
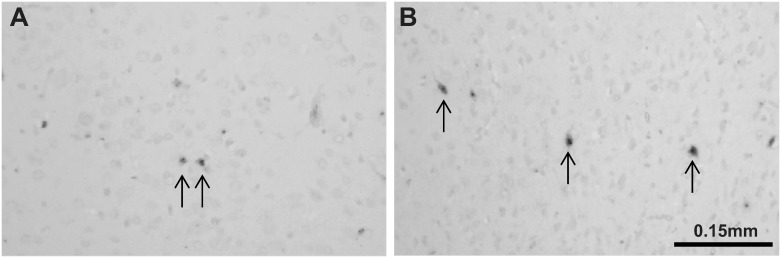
Representative TUNEL sections. At x20 magnification from the anesthesia group (A) and anesthesia + surgery group (B).

**Fig 2:**
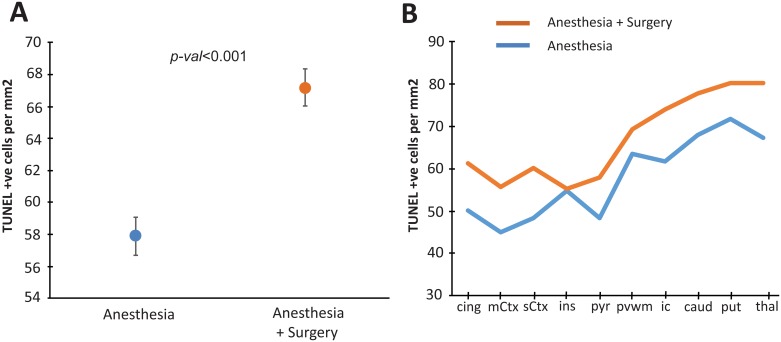
Quantification of TUNEL histology. There was an overall increase in the estimated mean TUNEL-positive cells per mm^2^ (pooled across regions and R0/ R1 levels) in the anesthesia + surgery group versus anesthesia alone. On regional assessment there was a significant increase in TUNEL positive cells in the anesthesia + surgery group versus anesthesia alone in the cingulate cortex (cing), motor cortex (mCtx), somatosensory cortex (sCtx), pyriform cortex (pyr), internal capsule (ic), caudate (caud) and thalamus (thal). No significant increases were observed in the insula cortex, periventricular white matter (pvwm) or putamen (put).

Cell death assessed by TUNEL staining and statistical results are illustrated in Figs 1 and 2 and Table 2. An increase in cell death was observed following surgery as an overall effect of treatment across all ten areas examined (<0.0001) (Figs 1 and 2, Table 2). Cell death in piglets who underwent 15 min of inguinal surgery was significantly higher than that in piglets exposed to isoflurane anaesthesia alone in the cingulate, motor, somatosensory and pyriform cortices, the internal capsule, caudate nucleus and thalamus (Figs 1 and 2, Table 2). In the insula cortex, periventricular white matter and putamen there were no significant increases in cell death in piglets who underwent surgery.

**Table 2:** Statistical details of TUNEL counts for all 9 regions and overall, cell death in 7 regions was increased in the anesthesia + surgery group versus anesthesia alone. Statistical significance is indicated in bold.

Brain area	Difference in mean TUNEL count	95% CI	*p- val*
Cingulate cortex	11.0	(3.7, 18.2)	**0.003**
Motor cortex	10.7	(34.0, 18.0)	**0.004**
Somatosensory cortex	12.0	(4.8, 19.0)	**0.001**
Insula cortex	0.4	(-22.2, 9.7)	0.938
Pyriform cortex	9.2	(2.0, 16.5)	**0.013**
Pvwm	5.7	(-1.3, 12.9)	0.113
Internal capsule	12.2	(2.9, 21.5)	**0.011**
Caudate	10.0	(2.7, 18.9)	**0.043**
Putamen	8.5	(-0.79, 17.8)	0.073
Thalamus	13.0	(3.6, 17.8)	**0.007**
All areas	9.80	(6.61, 13.0)	**0.001**

### 15 mins of surgery induces significant changes in the expression of 181 gene transcripts in the cingulate cortex

**Fig 3:**
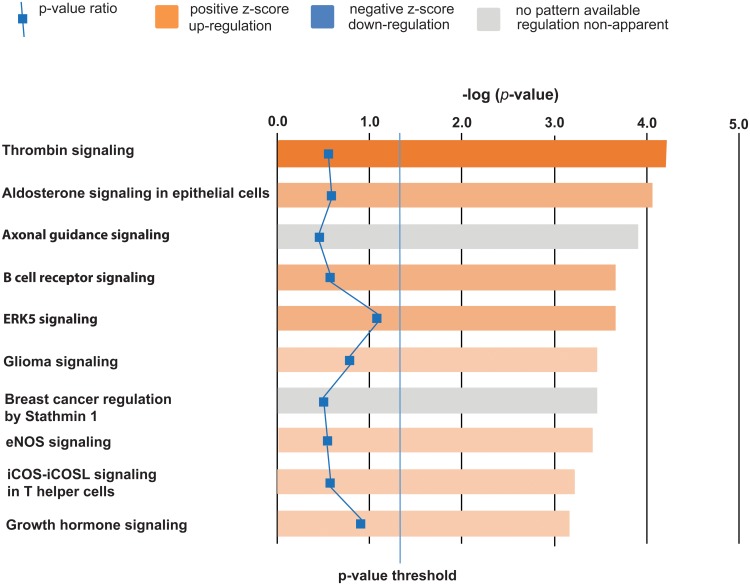
Pathway analysis illustrating the top ten canonical pathways affected by 15 mins of surgery. A positive z-score suggests an increase in pathway activity, the darker the orange colour the higher the increase in pathway activity. Our data suggests that surgery increases signalling in the thrombin, aldosterone, B-cell, ERK-5, eNOS, iCOS-iCOSL signalling in T-helper cells and growth hormone signalling pathways. A grey colour indicates that axon guidance signalling and breast cancer signalling by stathmin-1 is perturbed by surgery but its direction is not-apparent. Values are derived by the change in relative expression compared with the control group (fold change). This data suggests that surgery affects endothelial function, neural plasticity and inflammatory processes.

All gene expression data is reported as fold-change from the anaesthesia treatment group only. Surgery induced significant changes in the expression of 181 gene transcripts at a threshold of ±1.3 fold change, of these 163 gene transcripts were up-regulated and 18 were down-regulated (all 181 transcripts are available online as S1 Table and RNAseq data files are available as S1 Dataset). Analysis of functional significance using Ingenuity Pathway Analysis software demonstrated that surgery activated a number of pathways that may mediate adverse neural and cognitive development, the top 10 canonical pathways in order of significance are; thrombin signalling, aldosterone signalling in epithelial cells, axonal guidance signalling, B-cell receptor signalling, ERK-5 (or big MAP-kinase 1) signalling, glioma signalling, breast cancer regulation by stathmin 1, eNOS signalling, iCOS-iCOSL signalling in helper T-cells and growth hormone signalling. The top 10 pathways are illustrated in Fig 3 and the complete pathway analysis output is available online as S2 Table). Empirical observations of gene function also suggest that surgery also affected calcium signalling (7/181) and phosphoinosiditide function (6/181). We also observed that surgery induced changes in two gamma-amino-butyric acid type A subunits and the GABA-signalling pathway although this was not in the Top Ten pathways generated.

## Discussion

In a pre-clinical neonatal piglet model we demonstrate that 15 min of surgery completed within 1h of the induction of anesthesia, similar to an infant inguinal hernia repair in duration, location and area of tissue explored [17], increases cell death and induces significant changes in the expression of 181 gene transcripts in the anterior cingulate cortex when compared to a 6h exposure of 2% isoflurane and fentanyl (3mcg kg^-1^ h^-1^) alone. In combination with our prior data [10, 18], this finding indicates that animal models of “anesthesia alone” may be sub-optimal for assessing the likely consequences of exposure to anesthesia plus surgery, employed in human trials.

Whilst it is clearly established that early developmental exposure of the developing brain to commonly used general anesthesic agents *without surgery* induces significant increases in cell death in a range of animal species [1, 3, 4], this is the first observation that a brief surgical procedure increases cell death and induces transcriptional changes that are additional to those induced by exposure to anesthesia alone in a biologically relevant sample size of 12 animals per group. We have recently demonstrated that 6h exposure to isoflurane (in an identical manner to our control group) induces significant increases in the death of both neurons and oligodendrocytes [10]. The additional increases in cell death we observe following surgery are modest and although we have not carried out a quantitative double labelling study it is likely that the additional cell death we have observed also reflects the death of both neurons and oligodendrocytes.

Surgery even when accompanied by anesthesia is a sensory insult, which results in tissue damage and inflammation [19], stress [20] and developmentally inappropriate nocieceptive stimuli [18–21]. The piglets in our model, are less likely to have experienced “stress and pain” during the procedure than a human infant, as they did not have an underlying medical condition to act as a primary stressor, they were also sedated immediately on reception to our facility, were given fentanyl pain relief throughout and were unconscious throughout the procedure. They will have experienced a developmentally inappropriate nocieceptive stimuli from the surgery, but are unlikely to have experienced additional “stress and pain” either during the procedure or before. We chose the anterior cingulate to investigate transcriptional changes as it is important for the mediation of pain, anesthesia and cognitive function [22, 23, 24]. Our transcriptional data suggests a number of mechanisms that may contribute to an increase in cell death and exert adverse effects on neural or cognitive development. Our pathway analysis data suggests that a brief surgical procedure disrupts developmentally appropriate thrombin, aldosterone, B cell receptor, ERK-5, eNOS, growth hormone and iCOS-iCoSL helper T cell signalling in the brain by increasing activity in these pathways. It also suggests that surgery perturbs signalling in the axon guidance pathway in a manner that is neither, positive or negative (Fig 3 and S2 Table). A disruption to any or all of these signalling pathways may have pathological effects that may lead to developmentally inappropriate alterations in neural development and cognitive function. The thrombin signalling pathway is important in coagulation, normal neural development, vascular endothelial cell growth and the neural response to trauma [24, 25]. At low concentrations thrombin influences neuron and astrocyte development, induces glial cell proliferation and exerts a neuroprotective effect, however at high concentrations thrombin disrupts the function of the blood brain barrier and induces oedema and inflammation [26, 27]. Aldosterone signalling acts in neural epithelial tissues to mediate oxidative stress, inflammation, water and electrolyte homeostasis and is thought to be important in the pathophysiology of hypertension and stroke [28–30]. Developmentally and environmentally appropriate spatiotemporal control of axon growth and guidance is of crucial importance for the developmental remodelling and maturation of neural circuits and disruptions to this process may lead to a delay in the maturation of, and lifelong changes to neural function [31–34]. Disruption to B-cell signalling may affect a number of B-cell functions, including survival, apoptosis, proliferation, and differentiation into antibody-producing cells or memory B cells [35, 36]. The ERK-5 (aka big mitogen-activated protein kinase-1) signalling cascade is activated by a range of growth factors, cytokines and cellular stresses. Targeted inactivation of ERK-5 has also demonstrated that it is important for vascular integrity and in endothelial cells ERK-5 is required to restrain apoptosis, regulate hypoxia, tumour angiogenesis and cell migration [37, 38]. The role of eNOS signalling in the regulation of vascular tone, cellular proliferation, leukocyte adhesion and platelet aggregation is well established and its modification is thought to exert neuroprotective effects in a number of brain injury models [39–43]. iCOS-iCOSL signalling derived from helper T-cells provides the required signal to promote B-cell survival and functional maturation [44]. Both the growth hormone receptor and growth hormone itself is expressed widely in the brain and is thought to be important for neurogenesis, cell survival, myelin synthesis, dendritic branching and a range of cognitive functions [45–47]. Other changes observed include the upregulation of two GABA_A_ subunit transcripts and the GABA-A pathway, which is particularly salient given this pathway is a major target of anesthetic drugs [48] and that GABA_A_ receptor expression is regulated by inflammation [49, 50].

This genomic data needs further exploration in other model systems, but this data has important implications for the future development of neuroprotective strategies in neonates and infants undergoing anesthesia and surgery. Three themes arise from our genomic data that surgery induces an alteration in endothelial function (thrombin, aldosterone, ERK-5 and eNOS signalling) [24, 30, 37–43], inflammation (B-cell receptor, GABA-A and iCOS-iCOSL T-helper cell signalling) [35, 36, 44] and neural plasticity (axonal, GABA_A_ and growth hormone signalling) [31, 34, 45, 46, 48, 50]. Developmentally inappropriate perturbations to any of these processes have the potential to contribute to the development of adverse neural and cognitive development. Our results are also interesting and surprising in that whilst our changes in gene expression are modest, the surgery that induced them was a very brief 15 min procedure carried out 6h previously, it is likely that longer more complex surgical procedures (1h or so) will induce significantly more robust increases in both cell death and changes in gene expression supporting endothelial function, neural plasticity and inflammation, which could potentially contribute to a higher risk of adverse neural development. Of course it is also unclear whether these genomic changes are transient or not. For example, while this is unknown at present, long-term changes in GABA_A_ expression could contribute to the detrimental effects of repeat anesthesia exposure. Our genomic data is both unusual and robust in that we have employed biologically salient sample sizes of 12 animals per group for our RNAseq analysis, this is an important consideration as due to biological variability and the stochiastic nature of gene expression there is a debate concerning the importance of “read depth” versus “number of replicates” in accurately assessing the number of significantly differentially expressed genes independently of their fold change. It has been suggested due to these processes that 12 replicates is a minimum group size to detect the majority of these changes regardless of “read depth” [51, 52], however most studies only use 3–6 replicates. Our approach using 12 replicates has detected changes in the expression of 181 genes even after correction for repeated measures (using a Benjamini-Hochberg test). Our data does have some limitations as gene expression is a stochiastic and dynamic process and our data is derived from one 6h time point only, it would be interesting for example to do a longitudinal gene expression study at a number of time points following a surgical procedure, especially in the weeks following surgery but our data suggests multiple new avenues of enquiry for this field of research.

## Caveats associated with our study

Our study was designed as a succinct proof of concept study which clearly demonstrates that a brief period of surgery induces increases in cell death and changes in expression of genes important for neurodevelopment and cognitive function. Our study does have limitations in that we only sampled changes in cell death and gene expression at one 6h time point. It is therefore possible that cell death in both the control group and the group that underwent surgery would continue to increase for a 24h period and then decline. This conceivably could result in the convergence or divergence in the incidence of cell death between the two groups, which could affect the interpretation of our data. Our anesthesia treatment is also very long which does limit its clinical relevance as very few infants undergo 6h of anesthesia. Our piglets were also anesthetised throughout the procedure and were not allowed to reawaken following the surgical procedure, which may affect how our data in interpreted in relation to human studies. We also compared cell death and gene expression in male pigs only, this was a deliberate decision on our part as the effects of exposure to isoflurane and surgery may be sexually dimorphic, isoflurane in particular is known to increase brain cell death equivalently in both genders but induces cognitive deficits in males but not females [53]. This may be an important confounding factor when interpreting human data as some studies have suggested that neonatal anesthesia exposure is associated with attention deficit hyper-activity disorder [14], a pattern of cognitive development that is more common in males [54].

## Conclusions

The recent publication of preliminary cognitive data from the GAS consortium derived from comparing two groups of infants who were both exposed to a short surgical procedure, concluded that exposure to a single exposure of servoflurane anesthesia is not associated with an increased risk of adverse cognitive development at the age of 2 [17]. Our observations suggesting that surgery itself increases cell death adds an extra layer of complexity which may inform the interpretation of this and human studies that have attempted to link cognitive dysfunction to infantile exposure [11–17]. This suggests the need for further research into the mechanisms by which surgery itself may influence the development of the brain independently or synergistically with the effects of anaesthesia including how the severity and duration of both interact. Whilst the relationship between developmental anaesthesia exposure and the potential induction of cognitive deficits is complex and the subject of considerable debate, our data demonstrates that a brief surgical procedure increases cell death and alters the expression of genes important for endothelial function, inflammation and neural plasticity.

## Supporting information

S1 Table Gene transcripts responsive to 15 minutes of surgery. Surgery induced significant changes in the expression of 181 gene transcripts at a threshold of ±1.3 fold change, of these 163 gene transcripts were up-regulated and 18 were down-regulated. Analysis of gene expression was performed using a one-way ANOVA followed by a moderated T-test post-hoc test and a Benjami-Hochberg FDR multiple testing correction and *p*-values calculated asymptotically using endogenous Genespring GX12 software. A brief summary of function is included with each gene transcript.
(DOC)

S2 Table Canonical pathways represented by a brief 15 min period of surgery. Analysis was conducted using Ingenuity Pathway Analysis software on 181 gene transcripts expressed differentially in piglets exposed to a brief period of surgery.
(XLS)

S1 Dataset RNAseq datafiles. Each piglet has a separate sheet.
(XLS)
