# Supplementary material for: Surgery increases cell death and induces changes in gene expression compared with anesthesia alone in the developing piglet brain
Source: PLoS One. 2017 Mar 29;12(3):e0173413. doi: 10.1371/journal.pone.0173413 (PMC5371291; doi:10.1371/journal.pone.0173413)
Supplement: S1 Table — Surgery induced significant changes in the expression of 181 gene transcripts at a threshold of ±1.3 fold change, of these 163 gene transcripts were up-regulated and 18 were down-regulated. Analysis of gene expression was performed using a one-way ANOVA followed by a moderated T-test post-hoc test and a Benjami-Hochberg FDR multiple testing correction and p-values calculated asymptotically using endogenous Genespring GX12 software. A brief summary of function is included with each gene transcript. (DOC) [file pone.0173413.s001.doc]

**Supplementary Table 1**

**Surgery exacerbates cell death and induces changes in gene expression compared with anesthesia alone in the developing piglet brain.**

*Kevin D. Broad, PhD., *Go Kawano, MBBS, PhD., Igor Fierens, MBBS., Eridan Rocha-Ferreira, PhD., Mariya Hristova, PhD., Mojgan Ezzati, MD., Jamshid Rostami, MD., Daniel Alonso-Alconada, PhD., Badr Chaban, MBBS., Jane Hassell, MBBS., Bobbi Fleiss, PhD., Pierre Gressens MBBS, PhD., Robert D. Sanders, MBBS, PhD., Nicola J. Robertson, MB, ChB, PhD.

* Joint contribution.

| **Changes in the expression of 181 gene transcripts induced by surgery.**  **Details of all 181 gene transcripts.** | | | | | | |
| --- | --- | --- | --- | --- | --- | --- |
|  | | | | | | |
| **Fold**  **Change** | ***p-val*** | **All expression is relative to anesthesia exposure only** | **Abbreviation** | | **Accession where appropriate** | |
|  |  |  |  | |  | |
|  |  | **Downregulated** |  | |  | |
|  |  |  |  | |  | |
| **-1.59** | **0.012** | **Immunoglobin superfamily member 9** | **IGSF9** | | **XM_00355096** | |
|  |  | Dendrite outgrowth and synapse maturation | | | | |
| **-1.53** | **0.020** | **Retinoid X receptor** | **RXRG** | | **NM_001130211** | |
|  |  | Mediates the anti-proliferative effects of retinoic acid | | | | |
| **-1.45** | **0.009** | **Hekokinase 3 (white cell)** | **HK3** | |  | |
|  |  | Phosphorylates glucose to produce glucose 6-phosphate | | | | |
| **-1.38** | **0.012** | **Adhesion molecule, interacts with CXADR antigen 1** | **AMICA 1** | | **XM_003129904** | |
|  |  | Control of activation/ transmigration of leucocytes/ T cells | | | | |
| **-1.35** | **0.043** | **DnaJ (Hsp 40) homolog, subfamily B, member 13** | **DNAJB13** | | **NP_001182050** | |
|  |  | Molecular chaperone which mediates protein folding | | | | |
| **-1.34** | **0.039** | **Dead end microRNA, mediated expression inhibitor 1** | **DND 1** | |  | |
|  |  | Inhibits microRNA expression | | | | |
| **-1.34** | **0.046** | **Protocadherin 8** | **PCDH8** | | **XM_003131006** | |
|  |  | Mediates activity dependent synaptic re-organisation | | | | |
| **-1.33** | **0.001** | **Primase 2** | **PRIM 2** | | **NM_001244164** | |
|  |  | Synthesises small RNA primers during DNA replication | | | | |
| **-1.32** | **0.011** | **Four and a half LIM domains 2** | **FHL2** | |  | |
|  |  | Regulates transcriptional repressor E4F1, cell growth, apopotosis | | | | |
| **-1.32** | **0.048** | **Coiled coil domain containing 37** | **CCDC37** | | **XM_001924211** | |
|  |  | No confirmed function | | | | |
| **-1.31** | **0.016** | **Adaptor related protein complex 2** | **APIG2** | | **XM_001928659** | |
|  |  | **Gamma 2 subunit** | **G2AD** | |  | |
|  |  | Protein transport via transport vesicles | | | | |
| **-1.31** | **0.014** | **MHC class II regulatory factor** | **RFX1** | | **XM_003123351** | |
|  |  | Positive regulator of MHC class II gene | | | | |
| **-1.31** | **0.006** | **Collagen triple helix predicted repeat containing 1** | **CTHRC1** | | **XM_003125535** | |
|  |  | Mediates vascular remodelling following injury | | | | |
| **-1.31** | **0.014** | **Tox high mobility group box family member 3** | **TOX 3** | | **XM_003126986** | |
|  |  | Induces anti-apoptotic and represses pro-apoptotic transcripts | | | | |
| **-1.31** | **0.046** | **Matrix metallopeptidase II (stomelysin 3)** | **MMPII** | | **XM_001167795** | |
|  |  | **Stomelysin 3** | **STMY3** | |  | |
|  |  | Breakdown of extracellular matrix | | | | |
| **-1.31** | **0.008** | **Actin, alpha 1, skeletal muscle** | **ACTA1** | | **NM_001167795** | |
|  |  | Cell motility, structure and integrity | | | | |
| **-1.30** | **0.003** | **Calponin 1, basic smooth muscle** | **CNN1** | | **NM_213878** | |
|  |  | Modulation of smooth muscle contraction | | | | |
|  |  |  |  | |  | |
| **Upregulated** | | | | | | |
|  | | | | | | |
| **1.30** | **0.033** | **Leucine rich repeat containing 8 family member B** | **LRRC8B** | | **XM_00355301** | |
|  |  | Mediates volume-regulated anion channel | | | | |
| **1.30** | **0.032** | **Nuclear factor activated T cells, tonicity responsive** | **NFAT5** | |  | |
|  |  | Transcriptional regulation of osmo-protective/ inflammatory genes | | | | |
| **1.30** | **0.047** | **Calcium/calmodulin dependent protein kinase 4** | **CAMK4** | |  | |
|  |  | Modulation of calcium signalling following NMDA receptor activation | | | | |
| **1.30** | **0.045** | **GATA zinc finger domain containing 2B** | **GATAD2B** | |  | |
|  |  | Transcriptional repressor important in synapse development | | | | |
| **1.30** | **0.036** | **Striatin calmodulin binding protein** | **STRN** | | **XM_003125240** | |
|  |  | Modulates calcium signalling a scaffolding protein | | | | |
| **1.31** | **0.045** | **Pre B cell leukemia homeobox** | **PBX1** | | **XM_003355079** | |
|  |  | Regulation of osteogenesis, pre B-cell lymphoblastic leukaemia | | | | |
| **1.31** | **0.030** | **Low density lipoprotein receptor related protein 6** | **LRP6** | | **XM_003126472** | |
|  |  | Wnt/β-catenin signalling, cell differentiation, proliferation, migration | | | | |
| **1.31** | **0.041** | **Ataxia cerebellular Cayman type** | **ATCAY** | | **XM_003354012** | |
|  |  | Regulates glutamate production via glutaminase/ GLS enzyme, localisation of mitochondria within axons and dendrites | | | | |
| **1.31** | **0.017** | **Multiple inosityl polyphosphate phosphatase 1** | **MINPP1** | | **XM_001927672** | |
|  |  | Phosphatidylinositol signalling pathway-neuronal plasticity | | | | |
| **1.31** | **0.041** | **Dix domain containing 1** | **DIXDC1** | | **XM_003357296** | |
|  |  | Positive effector of the Wnt signalling pathway | | | | |
| **1.31** | **0.039** | **Fat storage inducing transmembrane protein 2** | **FITM2** | | **NM_001128460** | |
|  |  | Lipid droplet accumulation, cell morphology, cytoskeletal function | | | | |
| **1.31** | **0.004** | **Plexin domain containing 1** | **PLXDC1** | |  | |
|  |  | Regulates cell capillary morphogenesis | | | | |
| **1.31** | **0.010** | **Zinc finger protein 24** | **ZNF24** | | **NM_001243375** | |
|  |  | Transcription factor required for myelination of oligodendrocytes | | | | |
| **1.31** | **0.045** | **Inositol polyphosphate 4 phosphatase type 1, 107kDa** | **INPP4A** | | **NM_001143930** | |
|  |  | Phosphatidylinositol signalling pathway-neuronal plasticity | | | | |
| **1.31** | **0.035** | **Solute carrier family 35 member E1** | **SLC35E1** | | **XM_003123474** | |
|  |  | Transportation across cell membranes | | | | |
| **1.31** | **0.014** | **Helicase like transcription factor** | **HLTF** | | **XM_00338605** | |
|  |  | Transcriptional regulation by modulation of chromatin structure | | | | |
| **1.32** | **0.004** | **Fukutin** | **FKTN** | |  | |
|  |  | Putative transmembrane protein important for brain development | | | | |
| **1.32** | **0.043** | **ELKS/ RAB6 interacting CAST family member 2** | **ERC2** | | **XM_003358480** | |
|  |  | Active zone protein that regulates neurotransmitter release | | | | |
| **1.32** | **0.022** | **Zinc finger protein 398** | **ZNF 398** | | **XM_00313028** | |
|  |  | Kruppel family zinc finger transcription factor | | | | |
| **1.32** | **0.043** | **Phosphatidyl inositol transfer protein** | **PITPNC1** | | **NM_001143722** | |
|  |  | Phosphatidylinositol signalling pathway-neuronal plasticity | | | | |
| **1.32** | **0.029** | **Unc-80 homolog (Celegans)** | **UNC80** | | **XM_003133630** | |
|  |  | Component of the NALCN sodium channel complex a cation channel activated by substance P or neurotensin which controls neuronal excitability | | | | |
| **1.32** | **0.021** | **Amyloid beta precursor protein (cytoplasmic tail) binding protein 2** | **APPBP2** | | **XM_003131695** | |
|  |  | Associated with β-amyloid precursor protein transport | | | | |
| **1.32** | **0.022** | **Forkhead box N3** | **FOXN3** | | **NM_001044536** | |
|  |  | Forkhead/ winged transcription factor mediates eukaryotic dna-damage inducible cell cycle arrests at G1 and G2 | | | | |
| **1.32** | **0.039** | **Phosphatase and tensin homolog** | **PTEN** | | **NM_001143696** | |
|  |  | Phosphatidylinositol signalling pathway-neuronal plasticity | | | | |
| **1.32** | **0.013** | **Kelch like family member 24** | **KLHL24** | |  | |
|  |  | Reduces kainite receptor mediated currents in neurons |  | |  | |
| **1.32** | **0.034** | **Guanine nucleotide binding protein (G protein) alpha 13** | **GNA13** | | **XM_003357998** | |
|  |  | Modulation/ transduction of transmembrane signalling | | | | |
| **1.32** | **0.030** | **Potassium voltage gated channel, Shaw related subfamily, member 3** | **KCNC3** | | **XM_003356005** | |
|  |  | Voltage gated potassium channel neuronal excitability | | | | |
| **1.32** | **0.034** | **R3H domain containing 1** | **R3HDM1** | | **XM_003133267** | |
|  |  | Binds single stranded nucleic acids and poly (A) RNA binding | | | | |
| **1.33** | **0.017** | **Major facilitator superfamily domain containing 4** | **MFSD4** | | **XM_003357394** | |
|  |  | Membrane transport exact passenger unknown | | | | |
| **1.33** | **0.015** | **Solute carrier family 12 (sodium/ potassium/chloride transporter) member 2** | **SLC12A2** | | **XM_003123899** | |
|  |  | Membrane protein maintains ionic (Na^+^/K^+^/CL^-^) balance/ cell volume | | | | |
| **1.33** | **0.027** | **EH domain containing 4** | **EHD4** | | **XM_003480399** | |
|  |  | Membrane binding protein controls membrane reorganisation | | | | |
| **1.33** | **0.048** | **Ganglioside induced differentiation associated protein** | **GDAP1** | | **XM_001927060** | |
|  |  | Cell differentiation and neurite outgrowth | | | | |
| **1.33** | **0.049** | **Aldehyde dehydrogenase 1 family, member L2** | **ALDHIL2** | | **XM_001926587** | |
|  |  | Important gene in mitochondrial function (NADP^+^ dependent) | | | | |
| **1.33** | **0.047** | **Choroiderema (Rab export protein 1)** | **CHM** | |  | |
|  |  | Binds Rab-GTPases which co-ordinates vesicle traffic | | | | |
| **1.33** | **0.002** | **Zinc finger 256** | **ZNF256** | |  | |
|  |  | Kruppel family zinc finger protein | | | | |
| **1.33** | **0.039** | **Adenosyl homocysteinase-like 2** | **AHCYL2** | | **XM_003134679** | |
|  |  | Regulates sodium bicarbonate co-transporter SLC4A4 activity and Mg^2+^ sensitivity | | | | |
| **1.34** | **0.047** | **Solute carrier family 6 (neurotransmitter GABA transporter) member 1** | **SLC6A1** | | **XM_003483220** | |
|  |  | GABA transporter removes GABA from synaptic cleft | | | | |
| **1.34** | **0.036** | **Mannosyl (alpha 1-3) glycoprotein beta-1, 4-N acetylglucosaminyltransferase, isoenzyme A** | **MGAT4A** | **XM_003124907** | |  |
|  |  | Formation and transport of sugars | | | | |
| **1.34** | **0.036** | **Nuclear receptor subfamily 1, group D, member 2** | **NRID2** | |  | |
|  |  | Transcriptional repression of circadian genes (CLOCK), activates interleukin-6 | | | | |
| **1.34** | **0.041** | **Ras association (Ral/GDS/AF-6) domain family member 2** | **RASSF2** | | **XM_00335988** | |
|  |  | KRAS-specific protein may promote apoptosis/ cell cycle arrest | | | | |
| **1.34** | **0.045** | **Protein tyrosine phosphatase, non-receptor type 11** | **PTPN11** | | **XM_003359116** | |
|  |  | Mitogenic activation, metabolic control, transcriptional regulation, and cell migration |  | |  | |
| **1.34** | **0.005** | **Fibroblast growth factor receptor substrate 2** | **FRS2** | | **NM_001243209** | |
|  |  | Phosphatidylinositol signalling pathway-neuronal plasticity  Links FGR and NGR to downstream signalling pathways | | | | |
| **1.35** | **0.049** | **GRAM domain containing 1B** | **GRAMD1B** | |  | |
|  |  | Function unclear | | | | |
| **1.35** | **0.045** | **Ectonucleotide pyrophosphatase phosphodiesterase 5** | **ENPP5** | | **XM_001927534** | |
|  |  | Type-I transmembrane glycoprotein important for neuronal cell communication | | | | |
| **1.35** | **0.027** | **Ras association (Ral/ GDS/AF-6) and pleckstrin homology domains 1** | **RAPH1** | |  | |
|  |  | Negatively regulates cell adhesion | | | | |
| **1.35** | **0.028** | **Nuclear fragile X retardation protein interacting protein 2** | **NUFIP2** | | **XM_003131795** | |
|  |  | RNA binding and poly(A) binding | | | | |
| **1.35** | **0.014** | **Suppressor of cytokine signalling 4** | **SOCS4** | | **NM_001162889** | |
|  |  | Negative regulation of JAK/ STAT and cytokine signalling | | | | |
| **1.35** | **0.015** | **Arginyltransferase 1** | **ATE1** | |  | |
|  |  | Mediates ubiquitin degradation of proteins | | | | |
| **1.35** | **0.050** | **Kruppel like factor 13** | **KLF13** | | **NM_001011505** | |
|  |  | Represses transcription by binding to the RTE site, activates RANTES expression in T-cells | | | | |
| **1.36** | **0.034** | **Karyospherin (Importin alpha 7)** | **KPNA6** | | **XM_003356277** | |
|  |  | Adaptor protein for nuclear receptor immune system | | | | |
| **1.36** | **0.026** | **Phosphate cytidyltransferase 1, choline alpha** | **PCYT1A** | | **XM_003132613** | |
|  |  | Regulation of phosphatidylcholine biosynthesis | | | | |
| **1.36** | **0.043** | **Entonucleoside triphosphate diphosphohydrase 1** | **ENTPD1** | |  | |
|  |  | Plasma membrane protein hydrolyzes extracellular ATP/ ADP to AMP In the brain, regulation of purinergic neurotransmission | | | | |
| **1.36** | **0.017** | **Vacuolar protein sorting 8 homolog** | **VPS8** | |  | |
|  |  | Involved in the endosomal multivesicular bodies (MVB) pathway | | | | |
| **1.36** | **0.031** | **Zinc finger protein 671** | **ZNF671** | | **XM_003356103** | |
|  |  | Kruppel family zinc finger protein | | | | |
| **1.37** | **0.047** | **RAB11 family interacting protein 2 (class 1)** | **RAB11FIP2** | |  | |
|  |  | Phosphatidylinositol signalling pathway-neuronal plasticity | | | | |
| **1.37** | **0.041** | **Sema domain, immunoglobulin domain (Ig) short base domain secreted (semaphorin) 3E** | **SEMA3E** | | **XM_003130220** | |
|  |  | Regulate angiogenesis and axon guidance | | | | |
| **1.37** | **0.018** | **Adaptor related protein complex 3, mu2 subunit** | **AP3M2** | | **XM_003134229** | |
|  |  | It facilitates the budding of vesicles from the Golgi membrane | | | | |
| **1.37** | **0.036** | **Protein phosphatise, Mg2+/ Mn2+ dependent 1E** | **PPMIE** | |  | |
|  |  | **Ca(2+)/Calmodulin-Dependent Protein Kinase Phosphatase N** | **CAMKN** | |  | |
|  |  | Protein phosphatase that inactivates multifunctional CaM kinases such as CAMK4 and CAMK2 | | | | |
| **1.38** | **0.038** | **Interleukin 6 signal transducer** | **IL6ST** | | **NM_001097432** | |
|  |  | Signal transducer shared by many cytokines, including interleukin 6 (IL6), ciliary neurotrophic factor (CNTF), leukemia inhibitory factor (LIF), and oncostatin M (OSM). Essential for survival of motor/ sensory neurons and differentiation of astrocytes. | | | | |
| **1.38** | **0.018** | **Angomotin like 1** | **AMOTL1** | | **XM_003129771** | |
|  |  | Peripheral membrane protein component of tight junctions. Inhibits the Wnt/beta-catenin signalling pathway. | | | | |
| **1.38** | **0.027** | **Endoplasmic reticulum metallopeptidase 1** | **ERMP 1** | |  | |
|  |  | No obvious neural function | | | | |
| **1.38** | **0.025** | **Tubulin tyrosine ligase like family member 7** | **TTLL2** | | **XM_003482064** | |
|  |  | Required for neurite growth, responsible for the strong increase in tubulin polyglutamylation during postnatal neuronal maturation. | | | | |
| **1.38** | **0.044** | **E3 Ubiquitin protein ligase** | **UBRS** | | **XM_003125541** | |
|  |  | E3 ubiquitin-protein ligase that mediates ubiquitination and subsequent degradation of DVL1 | | | | |
| **1.38** | **0.038** | **Tubulin tyrosine kinase** | **TTL** | | **NM_001004041** | |
|  |  | Post translational modification of tubulin | | | | |
| **1.38** | **0.025** | **Kruppel like factor** | **KLF9** | | **NM_001011504** | |
|  |  | Transcriptional repressor and activator. | | | | |
| **1.38** | **0.007** | **Protein inhibitor of activated STAT1** | **PIAS1** | | **XM_003121753** | |
|  |  | Transcriptional co-regulation in diverse cellular pathways, including the STAT pathway, the p53 and the steroid hormone signalling pathway. | | | | |
| **1.39** | **0.030** | **Fem 1 homolog b (C.elegans)** | **FEM1B** | | **XM_003121751** | |
|  |  | Mediates apoptosis by acting as a death receptor-associated protein. | | | | |
| **1.39** | **0.033** | **Angiomotin** | **AMOT** | |  | |
|  |  | Expressed predominantly in endothelial cells mediates the migration of endothelial cells toward growth factors during the formation of new blood vessels. | | | | |
| **1.39** | **0.040** | **EGF repeats and discoiden 1 like domain 3** | **EDIL3** | | **XM_003123766** | |
|  |  | EGF stimulates the growth of epidermal and epithelial tissues. Can induce neurite outgrowth. | | | | |
| **1.39** | **0.046** | **Decapping mRNA2** | **DCP2** | |  | |
|  |  | Key component of an mRNA-decapping complex required for degradation of mRNAs. | | | | |
| **1.40** | **0.043** | **Sortilin 1** | **SORT1** | | **XM_003125872** | |
|  |  | Sorting receptor in the Golgi compartment and as a clearance receptor on the cell surface | | | | |
| **1.40** | **0.043** | **Attractin like 1** | **ALP** | |  | |
|  |  | May play a role in melanocortin signalling to regulate energy homeostasis. | | | | |
| **1.41** | **0.032** | **AF4/ FMR family member 4** | **AFF4** | |  | |
|  |  | Associated with the folate-sensitive fragile X E locus on chromosome X. | | | | |
| **1.41** | **0.031** | **Tyrosine phosphatise receptor type D** | **PTPRD** | | **XM_001924678** | |
|  |  | Signalling molecule that regulate a variety of cellular processes including cell growth, axon guidence, differentiation, mitotic cycle, and oncogenic transformation. | | | | |
| **1.41** | **0.038** | **Frizzled class receptor 3** | **FZD3** | | **XM_001928787** | |
|  |  | Receptor for Wnt proteins and coupled to the beta-catenin canonical signalling pathway. | | | | |
| **1.41** | **0.023** | **Required for meiotic nuclear division 5 homolog A (S.cerevisiae)** | **RMND5A** | |  | |
|  |  | Potential neural function unknown. | | | | |
| **1.41** | **0.038** | **Dep domain containing MTOR interacting protein 1** | **DEPTOR** | |  | |
|  |  | Negative regulator of the mTORC1 and mTORC2 signalling pathways. Regulates autophagy. | | | | |
| **1.41** | **0.048** | **Solute carrier family 4 sodium bicarbonate co transporter member 8** | **SLC4A8** | | **XM_003481580** | |
|  |  | Negative regulator of the mTORC1 and mTORC2 signalling pathways. Regulates autophagy. | | | | |
| **1.41** | **0.043** | **3’-phosphoinositide dependent protein kinase 1** | **PDPK1** | |  | |
|  |  | Serine/threonine protein kinase integral to the function of the PI 3-K/Akt signaling pathway. PDK1 and mTORC2 both phosphorylate and activate PKB/Akt, ensuring a cellular response to growth factors and insulin signalling. | | | | |
| **1.42** | **0.021** | **DNA damage inducible homolog 2 (S.cerevisiase)** | **DDI2** | |  | |
|  |  | Thought to function in DNA damage repair but specific neural function unknown. | | | | |
| **1.42** | **0.028** | **Mediator complex sub-unit 13** | **MED13** | | **XM_003483047** | |
|  |  | Regulates transcription of nearly all RNA polymerase II-dependent genes. | | | | |
| **1.42** | **0.036** | **Mothers against decapentaplegic homolog 3** | **SMAD3** | | **NP_999302** | |
|  |  | Intracellular signal transducer and transcriptional modulator activated by TGF-beta (transforming growth factor) and activin type 1 receptor kinases. | | | | |
| **1.43** | **0.024** | **Large tumour suppressor kinase 1** | **LATS1** | |  | |
|  |  | Involved in the control of p53 expression. | | | | |
| **1.43** | **0.033** | **AVL9 homolog (S.cerevisiase)** | **AVL9** | |  | |
|  |  | Functions in cell migration. | | | | |
| **1.43** | **0.040** | **Rho guanine nucleotide exchange factor (GEF) 12** | **ARHGEF12** | | **XM_003129956** | |
|  |  | Mediates cellular processes that are initiated by extracellular stimuli working through G protein-coupled receptors. | | | | |
| **1.43** | **0.022** | **Ubiquitin like modifier activating enzyme 6** | **UBA6** | | **XM_003129053** | |
|  |  | Activates ubiquitin by first adenylating its C-terminal glycine residue with ATP, essential for embryonic development. | | | | |
| **1.43** | **0.046** | **CUB and Sushi multiple domains 1** | **CSMD1** | |  | |
|  |  | Functions as a tumour suppressor. | | | | |
| **1.43** | **0.029** | **Trafficking protein kinesin binding 2** | **TRAK2** | | **XM_003133586** | |
|  |  | Regulates endosome-to-lysosome trafficking of membrane cargo, including EGFR. | | | | |
| **1.43** | **0.020** | **Zyg11B homolog (C.elegans)** | **ZYG11B** | | **XM_003482079** | |
|  |  | Acts as target recruitment subunit in the E3 ubiquitin ligase complex ZYG11B-CUL2-Elongin BC. | | | | |
| **1.44** | **0.020** | **Protein tyrosine phosphatise, non-receptor type 4 (megakaryocyte)** | PTPN4 | |  | |
|  |  | Interacts with glutamate receptor delta 2 and epsilon subunits, and is thought to play a role in signalling downstream of the glutamate receptors through tyrosine dephosphorylation. | | | | |
| **1.44** | **0.003** | **Keltch repeat and BTB (POZ) domain containing 3** | **KBTBD3** | | **XM_003357271** | |
|  |  | Contains protein binding motifs but specific neural function uncertain. | | | | |
| **1.44** | **0.041** | **Quaking Homolog, KH Domain RNA Binding** | **QKI** | |  | |
|  |  | RNA-binding protein, regulates pre-mRNA splicing, export of mRNAs, protein translation, and mRNA stability. Involved in myelinization and oligodendrocyte differentiation. | | | | |
| **1.44** | **0.031** | **Mannosyl (alpha-1, 6-)glycoprotein beta 1-6-N acetyl glucosyl aminotransferase** | **MGAT5** | |  | |
|  |  | Enzyme involved in the regulation of the biosynthesis of glycoprotein oligosaccharides. | | | | |
| **1.44** | **0.022** | **Lysyl oxidase** | **LOX** | |  | |
|  |  | Responsible for post-translational oxidative deamination of peptidyl lysine residues in precursors to fibrous collagen and elastin. May have a direct role in tumour suppression. | | | | |
| **1.44** | **0.027** | **Guanine nucleotide binding protein (G protein), Q polypeptide** | **GNAQ** | |  | |
|  |  | Regulates B-cell selection and survival, required to prevent B-cell-dependent autoimmunity. Regulates chemotaxis of BM-derived neutrophils and dendritic cells. | | | | |
| **1.44** | **0.024** | **Cytoplasmic polyadenylation element binding protein 3** | **CPEB3** | |  | |
|  |  | Highly [conserved](https://en.wikipedia.org/wiki/Conserved_sequence) [RNA](https://en.wikipedia.org/wiki/RNA)-binding [protein](https://en.wikipedia.org/wiki/Protein) that promotes the elongation of the [polyadenine tail](https://en.wikipedia.org/wiki/Polyadenylation) of [messenger RNA](https://en.wikipedia.org/wiki/Messenger_RNA). | | | | |
| **1.44** | **0.043** | **Seizure related homolog (mouse) like** | **SEZ6L** | | **XM_001924686** | |
|  |  | Contributes to specialized endoplasmic reticulum functions in neurons. | | | | |
| **1.45** | **0.028** | **Thrombospondin, type 1 domain containing 7A** | **THSD7A** | | **XM_00357451** | |
|  |  | Promotes endothelial cell migration and filopodia formation during angiogenesis via a FAK-dependent mechanism. | | | | |
| **1.45** | **0.033** | **Set domain containing (lysine methyltransferase) 7** | **SETD7** | |  | |
|  |  | Histone methyltransferase that specifically monomethylates Lys-4 of histone H3. Plays a central role in the transcriptional activation of genes such as collagenase or insulin. | | | | |
| **1.46** | **0.021** | **Regulator of G protein signalling 7 binding protein** | **RGS7BP** | |  | |
|  |  | Binds to all members of the R7 subfamily of regulators of G protein signalling and regulates their translocation between the nucleus and the plasma membrane. | | | | |
| **1.46** | **0.032** | **Mannosidase alpha class 1A** | **MAN1A2** | |  | |
|  |  | This gene encodes a class I mammalian Golgi 1, 2-mannosidase which is a type II transmembrane protein. | | | | |
| **1.46** | **0.026** | **Protocadherin 7** | **PCDH7** | |  | |
|  |  | The gene product is an integral membrane protein that is thought to function in cell-cell recognition and adhesion. |  | |  | |
| **1.47** | **0.016** | **F box protein 32** | **FBX032** | | **NM_001044588** | |
|  |  | Substrate recognition component of a SCF (SKP1-CUL1-F-box protein) E3 ubiquitin-protein ligase complex which mediates the ubiquitination and subsequent proteasomal degradation of target proteins | | | | |
| **1.48** | **0.021** | **Gamma aminobutyric acid (GABA^A^receptor) gamma 2** | **GABRG2** | | **XM_003134087** | |
|  |  | GABA is the major inhibitory neurotransmitter in the brain, where it acts at GABA-A receptors, (ligand-gated chloride channels). | | | | |
| **1.48** | **0.019** | **Solute carrier family 13 (sodium dependent citrate transporter) member 5** | **SLC13A5** | |  | |
|  |  | High-affinity sodium/citrate cotransporter that mediates citrate entry into cells. | | | | |
| **1.48** | **0.033** | **Semaphorin 5A** | **SEMA5A** | |  | |
|  |  | Involved in axonal guidance during neural development (autism susceptibility gene). | | | | |
| **1.48** | **0.029** | **Hect, C2 and WW domain containing E3 ubiquitin protein ligase 2** | **HECW2** | | **XM_003483708** | |
|  |  | E3 ubiquitin-protein ligase that mediates ubiquitination of TP73. | | | | |
| **1.48** | **0.009** | **Transforming, acidic coiled coil containing protein 1** | **TACC1** | | **XM_003359484** | |
|  |  | Involved in the processes that promote cell division prior to the formation of differentiated tissues. | | | | |
| **1.48** | **0.020** | **Post GPI attachement to proteins 1** | **PGAP1** | | **XM_003359598** | |
|  |  | Involved in inositol deacylation of GPI-anchored proteins. This may be important for efficient transport of GPI-anchored proteins from the endoplasmic reticulum to the Golgi. | | | | |
| **1.49** | **0.032** | **ST8 alpha N acetyl neuramide alpha 2, 8 sialyltransferase 3** | **ST8SIA3** | |  | |
|  |  | Catalyzes the transfer of sialic acid from a CMP-linked sialic acid donor onto the terminal sialic acid of an acceptor through alpha-2,8-linkages. | | | | |
| **1.49** | **0.050** | **Kinesin family member 1B** | **KIF1B** | |  | |
|  |  | A motor protein that transports mitochondria and synaptic vesicle precursors. | | | | |
| **1.49** | **0.036** | **Fibronectin type III and SPRY domain containing 1 like** | **FSD1L** | |  | |
|  |  | Protein associates with a subset of microtubules and may be involved in the stability and organization of microtubules during cytokinesis. | | | | |
| **1.49** | **0.035** | **Potassium inwardly rectifying channel, sub family J member 3, transcript variant 1** | **KCNJ3** | | **XM_003483648** | |
|  |  | This protein is an integral membrane protein and inward-rectifier type potassium channel. | | | | |
| **1.50** | **0.034** | **Solute carrier family 38 member 1** | **SLC38A1** | |  | |
|  |  | Supplies glutamatergic and GABAergic neurons with glutamine which is required for the synthesis of the neurotransmitters glutamate and GABA. | | | | |
| **1.50** | **0.033** | **ADAM metallopeptidase domain 22** | **ADAM 22** | |  | |
|  |  | Probable ligand for integrin in the brain. Involved in regulation of cell adhesion and spreading and in inhibition of cell proliferation. Neuronal receptor for LGI1. | | | | |
| **1.50** | **0.019** | **Eukarotic translation inhibition factor 2C, 2** | **EIF2C2** | | **NM_001194975** | |
|  |  | **Argonute RISC catalytic component 2** |  | |  | |
|  |  | Required for RNA-mediated gene silencing (RNAi) by the RNA-induced silencing complex (RISC). | | | | |
| **1.50** | **0.024** | **Matrix metallopeptidase 16 (membrane inserted)** | **MMP16** | |  | |
|  |  | Involved in the breakdown of extracellular matrix in normal physiological processes, such as embryonic development, reproduction, and tissue remodelling. | | | | |
| **1.50** | **0.002** | **Amylo-alpha-1,1 6 glucosidase, 4 alpha glucanotransferase** | **AGL** | |  | |
|  |  | Glycogen debrancher enzyme which is involved in glycogen degradation. | | | | |
| **1.51** | **0.043** | **Calcium channel, voltage dependent alpha 2, delta subunit 1** | **CACNA2D1** | | **NM_214183** | |
|  |  | Plays an important role in excitation-contraction coupling. Molecular targets of gabapentin and pregabalin which are used to treat seizures and neuropathic pain. | | | | |
| **1.51** | **0.035** | **Discoidin domain receptor tyrosine kinase 2** | **DDR2** | |  | |
|  |  | Cell surface receptor for fibrillar collagen and regulates cell differentiation, remodelling of the extracellular matrix, cell migration and cell proliferation. | | | | |
| **1.51** | **0.036** | **Praja ring finger 2** | **PJA2** | | **XM_003483397** | |
|  |  | Has E2-dependent E3 ubiquitin-protein ligase activity. Essential for PKA-mediated long-term memory processes. | | | | |
| **1.51** | **0.015** | **Centrosomal protein 350kDa** | **CEP350** | |  | |
|  |  | Important for centriole growth by stabilizing a procentriolar seed composed of, SASS6 and CENPJ. | | | | |
| **1.51** | **0.031** | **Homeobox containing 1** | **HMBOX1** | |  | |
|  |  | Isoform 1 acts as a transcriptional repressor. | | | | |
| **1.51** | **0.034** | **Heat shock 70kDa protein 12A** | **HSPA12A** | |  | |
|  |  | Involved in the [ubiquitin](https://en.wikipedia.org/wiki/Ubiquitin)-[proteasome](https://en.wikipedia.org/wiki/Proteasome) pathway through interaction with the [AU-rich element RNA-binding protein 1](https://en.wikipedia.org/wiki/AUF1). Inhibits apoptosis. | | | | |
| **1.52** | **0.050** | **Mitogen activated protein kinase-kinase 9** | **MAP3K9** | |  | |
|  |  | This kinase blocks the ubiquitination of tumour suppressor p53, and increases the stability of p53 in non-stressed cells. | | | | |
| **1.53** | **0.023** | **LMBR1 Domain Containing 2** | **LMBRD2** | |  | |
|  |  | Probable lysosomal cobalamin transporter. | | | | |
| **1.53** | **0.039** | **Tau tubulin kinase 2** | **TTBK2** | | **XM_00335338** | |
|  |  | Phosphorylates tau and tubulin proteins. Mutations in this gene cause spinocerebellar ataxia type 11 (SCA11); a neurodegenerative disease characterized by progressive ataxia and atrophy. | | | | |
| **1.54** | **0.026** | **solute carrier family 30 (zinc transporter) member 4** | **SLC20A4** | | **NP_001124444** | |
|  |  | Probably involved in zinc transport out of the cytoplasm, by sequestration into an intracellular compartments. | | | | |
| **1.54** | **0.031** | **Calcitonin receptor like** | **CALCRL** | | **NM_241095** | |
|  |  | Receptor for calcitonin-gene-related peptide (CGRP) together with RAMP1 and receptor for adrenomedullin together with RAMP3. Receptor for adrenomedullin together with RAMP2. | | | | |
| **1.54** | **0.048** | **Tropomodulin 2 (neuronal)** | **TMOD2** | | **XM_001925676** | |
|  |  | Blocks the elongation and depolymerization of the actin filaments and contributes to the formation of the short actin protofilament, which in turn defines the geometry of the membrane skeleton. | | | | |
| **1.54** | **0.045** | **Storkhead box 2** | **STOX2** | | **XM_001925419** | |
|  |  | Differentially expressed in decidual tissue, involved in the susceptibility to pre-eclampsia with fetal growth restriction. | | | | |
| **1.55** | **0.025** | **ABL proto oncogene 2, non-receptor tyrosine kinase** | **ABL2** | |  | |
|  |  | Cell growth and survival such as cytoskeleton remodelling in response to extracellular stimuli, cell motility and adhesion and receptor endocytosis. | | | | |
| **1.55** | **0.039** | **SRY (Sex Determining Region Y)-Box 5** | **SOX5** | |  | |
|  |  | Encodes a member of the SOX (SRY-related HMG-box) family of transcription factors involved in the regulation of embryonic development and in the determination of the cell fate. | | | | |
| **1.55** | **0.030** | **Kinesin family member 5C** | **KIF5C** | | **XM_003359416** | |
|  |  | Mediates dendritic trafficking of mRNAs. | | | | |
| **1.55** | **0.039** | **Guanylate cyclase 1** | **GUCY1A2** | | **XM_003130093** | |
|  |  | Catalyzes the conversion of GTP to 3', 5'-cyclic GMP and pyrophosphate. This interacts with a beta subunit to form the guanylate cyclase enzyme, which is activated by nitric oxide. | | | | |
| **1.56** | **0.005** | **Na^+^/K^+^ transporting ATPase interacting 3** | **NKAIN3** | |  | |
|  |  | Important for sodium/ potassium transport in and out of cells. | | | | |
| **1.57** | **0.025** | **Protein kinase C, beta** | **PRKCB** | | **XM_003124545** | |
|  |  | This protein kinase has been reported to be involved in many different cellular functions, such as B cell activation, apoptosis induction, endothelial cell proliferation, and intestinal sugar absorption. | | | | |
| **1.57** | **0.050** | **Unc-13 homolog C (C.elegans)** | **UNC13C** | | **XM_003121500** | |
|  |  | Involved in the regulation of synaptic transmission at parallel fibres - Purkinje cell synapses | | | | |
| **1.57** | **0.024** | **Solute carrier family 23 (ascorbic acid transporter)** | **SLC23A2** | |  | |
|  |  | Important for vitamin C transport involving epithelial/ endothelial surfaces. | | | | |
| **1.57** | **0.025** | **Activating transcription factor 6** | **ATF6** | |  | |
|  |  | This gene encodes a transcription factor that activates target genes for the unfolded protein response (UPR) during endoplasmic reticulum (ER) stress. | | | | |
| **1.58** | **0.020** | **Calcium/ calmodulin dependent protein kinase 1D** | **CAMK1D** | |  | |
|  |  | Important in the CaMKK-CaMK1 signaling cascade and, upon calcium influx, activates CREB-dependent gene transcription, regulates calcium-mediated granulocyte function and promotes basal dendritic growth of hippocampal neurons. | | | | |
| **1.58** | **0.008** | **ETS domain protein (SRF accessory protein 1)** | **ELK4** | |  | |
|  |  | Transcription factor, important for the control of cytokine and chemokine genes. May control the differentiation, survival and proliferation of lymphoid cells. Regulates angiogenesis via regulation of expression of genes controlling endothelial cell migration and invasion. | | | | |
| **1.58** | **0.031** | **Leucyl/ cystinyl aminopeptidase** | **LNPEP** | | **NM_001105291** | |
|  |  | Degrades peptide hormones, oxytocin, vasopressin and angiotensin III. May be involved in the inactivation of neuronal peptides in the brain. Cleaves Met-enkephalin and dynorphin. Binds angiotensin IV and may be the angiotensin IV receptor in the brain. | | | | |
| **1.59** | **0.046** | **Synaptic vesicle glycoprotein 2B** | **SV2B** | | **XM_001926765** | |
|  |  | Probably plays a role in the control of regulated secretion in neural and endocrine cells. | | | | |
| **1.59** | **0.038** | **RAR related orphan receptor A** | **RORA** | |  | |
|  |  | Transcriptional regulation of genes involved in circadian rhythm. | | | | |
| **1.60** | **0.021** | **Clock circadian regulator** | **CLOCK** | | **XM_003356944** | |
|  |  | Transcriptional activator which forms a core component of the circadian clock. | | | | |
| **1.61** | **0.045** | **Kinase suppressor of Ras2** | **KSR2** | |  | |
|  |  | Acts as a negative regulator of MAP3K3-mediated activation of ERK, JNK and NF-kappa-B pathways, inhibiting MAP3K3-mediated interleukin-8 production. | | | | |
| **1.61** | **0.014** | **Sestrin 3** | **SESN3** | |  | |
|  |  | Induced by the p53 tumour suppressor protein and play a role in the cellular response to DNA damage and oxidative stress. Plays a critical role in antioxidant defence by regenerating overoxidized peroxiredoxins, the expression of this gene is a potential marker for exposure to radiation. | | | | |
| **1.62** | **0.043** | **Fat atypical cadherin 3** | **FAT3** | | **XM_003357241** | |
|  |  | Plays a role in the interactions between neurites derived from specific subsets of neurons during development. | | | | |
| **1.62** | **0.027** | **Ethanolamine phosphotransferase** | **EPT1** | | **XM_003481273** | |
|  |  | Catalyzes both phosphatidylcholine and phosphatidylethanolamine biosynthesis from CDP-choline and CDP-ethanolamine, respectively. | | | | |
| **1.63** | **0.040** | **alpha thalassemia/mental retardation syndrome X-linked** | **ATRX** | |  | |
|  |  | This protein is found to undergo cell cycle-dependent phosphorylation, which regulates nuclear matrix and chromatin association, and suggests its involvement in the gene regulation at interphase and chromosomal segregation in mitosis. | | | | |
| **1.63** | **0.017** | **Protein prenyltransferase alpha sub-unit repeat containing 1** | **PTAR1** | |  | |
|  |  | Catalyses lipidation which is essential for the biological function of a number of eukaryotic proteins, many of which are involved in signal transduction and vesicular traffic regulation. | | | | |
| **1.63** | **0.014** | **syntaxin binding protein 5-like** | **STXBP5L** | |  | |
|  |  | Important for vesicle trafficking and exocytosis. | | | | |
| **1.64** | **0.028** | **Bone morphogenetic protein receptor type II (serine/ threonine kinase)** | **BMPR2** | | **NM_001204900** | |
|  |  | Forms a receptor complex consisting of two type II and two type I transmembrane serine/threonine kinases. Type II receptors phosphorylate and activate type I receptors which autophosphorylate, then bind and activate SMAD transcriptional regulators. | | | | |
| **1.65** | **0.019** | **Suppressor of cancer cell invasion** | **SCAI** | | **XM_003122166** | |
|  |  | This gene encodes a regulator of cell migration. | | | | |
| **1.67** | **0.030** | **Solute carrier family 9 sub family A (NFIE7, cation proton antiporter 7) member 7** | **SLC9A7** | | **XM_003135064** | |
|  |  | This gene encodes a sodium and potassium/ proton antiporter that is a member of the solute carrier family 9 protein family. The encoded protein is primarily localized to the trans-Golgi network and is involved in maintaining pH homeostasis. | | | | |
| **1.72** | **0.044** | **Protein phosphatase 1 regulatory (inhibitor) sub-unit 9A** | **PPP1R9A** | | **XM_003357446** | |
|  |  | Imprinted, and located in a cluster of imprinted genes on chromosome 7q12. Transcribed in both neuronal and multiple embryonic tissues, maternally expressed in embryonic skeletal muscle tissues and biallelically expressed in other embryonic tissues. Controls actin cytoskeleton re-organisation. | | | | |
| **1.75** | **0.029** | **sacsin molecular chaperone** | **SACS** | |  | |
|  |  | Co-chaperone acts as a regulator of the Hsp70 chaperone machinery, involved in the processing of other ataxia-linked proteins. | | | | |
| **1.75** | **0.025** | **Cholinergic receptor muscarinic 3** | **CHRM3** | |  | |
|  |  | Influences the effects of acetylcholine in the central and peripheral nervous system. | | | | |
| **1.77** | **0.035** | **Cyclin dependent kinase like 5** | **CDKL5** | |  | |
|  |  | Regulates several neuronal development and physiological processes including neuronal survival, migration and differentiation, axonal and neurite growth, synaptogenesis, oligodendrocyte differentiation, synaptic plasticity and neurotransmission. | | | | |
| **1.78** | **0.024** | **synaptotagmin XVI** | **SYT16** | |  | |
|  |  | May be involved in the trafficking and exocytosis of secretory vesicles in non-neuronal tissues. Is Ca ^(2+)^ independant. | | | | |
| **1.79** | **0.025** | **Gamma aminobutyric acid A receptor beta 2** | **GABRB2** | |  | |
|  |  | Mediates inhibitory synaptic transmission in the central nervous system. This gene encodes GABA A receptor, beta 2 subunit.  NB GABA transmission may be excitatory during early development. | | | | |
| **1.82** | **0.011** | **Peroximal biogenesis factor 5 like** | **PEX5L** | | **XM_003132551** | |
|  |  | Plays an essential role in peroxisomal protein import | | | | |
| **2.12** | **0.025** | **Solute carrier family 24 (sodium/ potassium/ calcium exchanger) member 2** | **SLC24A2** | | **NM_001244595** | |
|  |  | Belongs to the SLC24 branch of exchangers, which mediate the extrusion of one Ca2+ ion and one K+ ion in exchange for four Na+ ions. | | | | |

**Supplementary Table 1. Gene transcripts responsive to 15 minutes of surgery.**

Surgery induced significant changes in the expression of 181 gene transcripts at a threshold of ±1.3 fold change, of these 163 gene transcripts were up-regulated and 18 were down-regulated. Analysis of gene expression was performed using a one-way ANOVA followed by a moderated T-test post-hoc test and a Benjami-Hochberg FDR multiple testing correction and *p*-values calculated asymptotically using endogenous Genespring GX12 software. A brief summary of known function is included with each gene transcript.
